# dFRAME: A Video Recording-Based Analytical Method for Studying Feeding Rhythm in *Drosophila*

**DOI:** 10.3389/fgene.2021.763200

**Published:** 2021-10-15

**Authors:** Mengxia Niu, Xiaohang Zhang, Weihan Li, Jianxun Wang, Yan Li

**Affiliations:** ^1^ School of Life Sciences, Beijing University of Chinese Medicine, Beijing, China; ^2^ Institute of Biophysics, State Key Laboratory of Brain and Cognitive Science, Center for Excellence in Biomacromolecules, Chinese Academy of Sciences, Beijing, China; ^3^ College of Life Sciences, University of Chinese Academy of Sciences, Beijing, China

**Keywords:** feeding rhythms, *Drosophila*, video recording, food approaching events, residence time, *period* mutant fly

## Abstract

Animals, from insects to humans, exhibit obvious diurnal rhythmicity of feeding behavior. Serving as a genetic animal model, *Drosophila* has been reported to display feeding rhythms; however, related investigations are limited due to the lack of suitable and practical methods. Here, we present a video recording-based analytical method, namely, *Drosophila* Feeding Rhythm Analysis Method (dFRAME). Using our newly developed computer program, FlyFeeding, we extracted the movement track of individual flies and characterized their food-approaching behavior. To distinguish feeding and no-feeding events, we utilized high-magnification video recording to optimize our method by setting cut-off thresholds to eliminate the interference of no-feeding events. Furthermore, we verified that this method is applicable to both female and male flies and for all periods of the day. Using this method, we analyzed long-term feeding status of wild-type and *period* mutant flies. The results recaptured previously reported feeding rhythms and revealed detailed profiles of feeding patterns in these flies under either light/dark cycles or constant dark environments. Together, our dFRAME method enables a long-term, stable, reliable, and subtle analysis of feeding behavior in *Drosophila*. High-throughput studies in this powerful genetic animal model will gain great insights into the molecular and neural mechanisms of feeding rhythms.

## Introduction

Feeding rhythm has recently been found to play a critical role in animal health. It is vital for animals to synchronize their feeding behavior to both internal biological clock and external environmental conditions, while the underlying mechanisms remain to be elucidated. With powerful tools for genetic and neural manipulation, *Drosophila melanogaster* serves as an excellent model for investigating the molecular and neural mechanisms underlying circadian rhythms (Franco et al., 2018). Earlier studies in *Drosophila* uncovered the molecular mechanisms controlling internal clock, which are evolutionarily conserved from insects to mammals (Yu and Hardin, 2006). Among the various environmental cues, light and food are the two major zeitgebers (Tomioka and Matsumoto, 2010; Barber et al., 2016; Challet, 2019; Helfrich-Forster, 2020). The sleep rhythm and the role of light entrainment have been well studied in flies (Shafer and Keene, 2021). In contrast, studies on feeding rhythms remain limited, largely because of the limitation of available analytical methods of fly feeding.

Various assays have previously been developed to study feeding behavior in fruit flies. One traditional category utilizes different types of tracers, such as non-digestible dyes (Edgecomb et al., 1994; Xu et al., 2008), radioactive substances (Thompson et al., 1991; Carvalho et al., 2005; Carvalho et al., 2006; Deshpande et al., 2014), and DNA oligomers in the BARCODE assay (Park et al., 2018; Huang et al., 2020). To study feeding rhythms using this approach, different groups of flies are transferred into food with tracers at different time points of the day. After a short period, usually less than 0.5 h to avoid excretion, the flies are quickly frozen, and the tracers in fly abdomens are quantified as the amount of food consumption. In these assays, normal laboratory food is used, and flies consume food in a natural manner. However, feeding behavior is inevitably being interfered by frequent manipulation, especially at night, and measurements are inevitably discontinuous.

The Capillary Feeder (CAFE) assay constitutes a practical way to continuously monitor food intake for several days (Ja et al., 2007; Diegelmann et al., 2017). In the CAFE assay, a small group of flies are put into a breeding vial, and they descend along a capillary and suck liquid food from it. The declining surface measured in the capillary represents the food amount consumed by the flies. Murphy et al. (2017) introduced an infrared monitor to the CAFE system, namely, Activity Recording Capillary Feeder (ARC), so that the food intake of single flies can be continuously recorded with a greatly improved sample rate of 1 min (Murphy et al., 2017; Li et al., 2021). Nevertheless, it is still difficult to capture single feeding events at this temporal resolution, therefore missing detailed information of feeding behavior. Moreover, the volatilization, stickiness, and potential leakage of liquid foods require strict control of the recording environment.

Proboscis extension response (PER) assay is utilized to study taste behavior in fruit flies, which is related to eating motivation (Wong et al., 2009). In the traditional setup, flies are fixed in a pipette tip, and upon food touching, their PER are monitored, however, for no longer than half an hour. To record naturally feeding flies, the Fly Proboscis and Activity Detector (fly PAD) was developed, which detects a capacitance signal whenever the proboscis of a free-moving fly touches food (Itskov et al., 2014). A similar assay called the Fly Liquid-Food Interaction Counter (FLIC) uses liquid food, and an analog electrical signal is measured (Ro et al., 2014). These two methods allow for continuous monitoring of feeding behavior in a high temporal resolution. However, the limited food amount used in these systems and the susceptibility to electromagnetic disturbances render long-term operation a considerable challenge.

*Drosophila* video recording system is a stable and easy-operating system, and it has been widely used to continuously monitor locomotor activity and study circadian rhythms of sleep (Zimmerman et al., 2008; Gilestro and Cirelli, 2009; Pfeiffenberger et al., 2010). Based on the image data obtained from this system, we designed a computer program called FlyFeeding to analyze food-approaching behavior. By setting filters, we distinguished food-approaching events (FAE) with feeding from those with no-feeding and determined three indexes, food-approaching events (FAEn), residence time of FAE (FAErt), and FAErt per event (FAErt/n). Three wild-type strains and a clock gene mutant were examined under fed/starvation and light–dark (LD)/dark–dark (DD) conditions using this analytical method. The results recaptured earlier reported features of feeding rhythms and, moreover, revealed new profiles of feeding patterns of these flies. Thus, our method, *Drosophila* Feeding Rhythm Analysis Method (dFRAME), allows for a stable, reliable, and extensive study of feeding rhythm in *Drosophila*.

## Materials and Equipment

### Fly Strains and Rearing

Fly strains were obtained from the Bloomington *Drosophila* Center, including the three wild-type strains *w* ^*1118*^, Canton S (CS), and *w*CS and a *period* mutant strain *period* ^*01*^ (*per* ^*01*^). Flies were reared on standard corn meal food (Bloomington recipe) and maintained at 25°C with 60% humidity on a 12-h/12-h LD cycle or constant darkness (DD). Wild-type *w* ^*1118*^ male flies were used for experiments unless otherwise specified.

### Experimental Setup and Video Recording

The *Drosophila* video recording system was set up according to previous reports (Pfeiffenberger et al., 2010; Gilestro, 2012). Briefly, individual flies at age of 3–5 days were transferred into a thin and straight glass tube with 5-mm diameter and 65-mm length. At one end of the tube, 2% agar with or without 5% sucrose was supplied as food. Flies were allowed to adapt to the environment for at least 12 h, and data collected thereafter were used for analysis. Images were recorded at one frame per second (fps) for 2–5 days, and the raw image data were processed to obtain the coordinate information of fly barycenter in each frame using pySolo (Gilestro and Cirelli, 2009). If the individual fly was not moving at the beginning of a day, the coordinate value of the first second of ZT 0 was automatically set to 0 (at the cotton side), and these traces were removed from the movement tracks.

### High-Magnification Video Recording

A high-magnification video recording system was constructed to characterize the food-approaching behavior. Individual flies in glass tubes were arranged under a Nikon SMZ25 stereomicroscope equipped with an ANDOR CCD camera. The area close to the food surface was video recorded at 5 fps for 30 min using the ANDOR Solis software. Videos were taken at five time-points of the day; for each time-point, the recording was repeated for at least four times. Data were exported in MP4 format prior to manual inspection.

### Statistical Analysis

All experimental data were analyzed and plotted with SPSS Statistics, GraphPad Prism, and Adobe Illustrator. *T* test (two-tailed, independent samples) was used to compare the two groups. One-way ANOVA and two-way ANOVA with Tukey’s honestly significant difference (Tukey HSD) *post hoc* test were applied to determine the difference among groups. Significance levels were set at *p* < 0.05 for all comparisons. Data were presented as mean ± standard error of mean (SEM). The following levels of significance were used: * or #, *p* < 0.05; ** or ##, *p* < 0.01; *** or ###, *p* < 0.001; **** or ####, *p* < 0.0001. n. s. indicates no significant difference. *n* represents the repetition of independent samples.

## Methods

### Food-Approaching Behavior Observed in the Video Recording System

**FIGURE 1:**
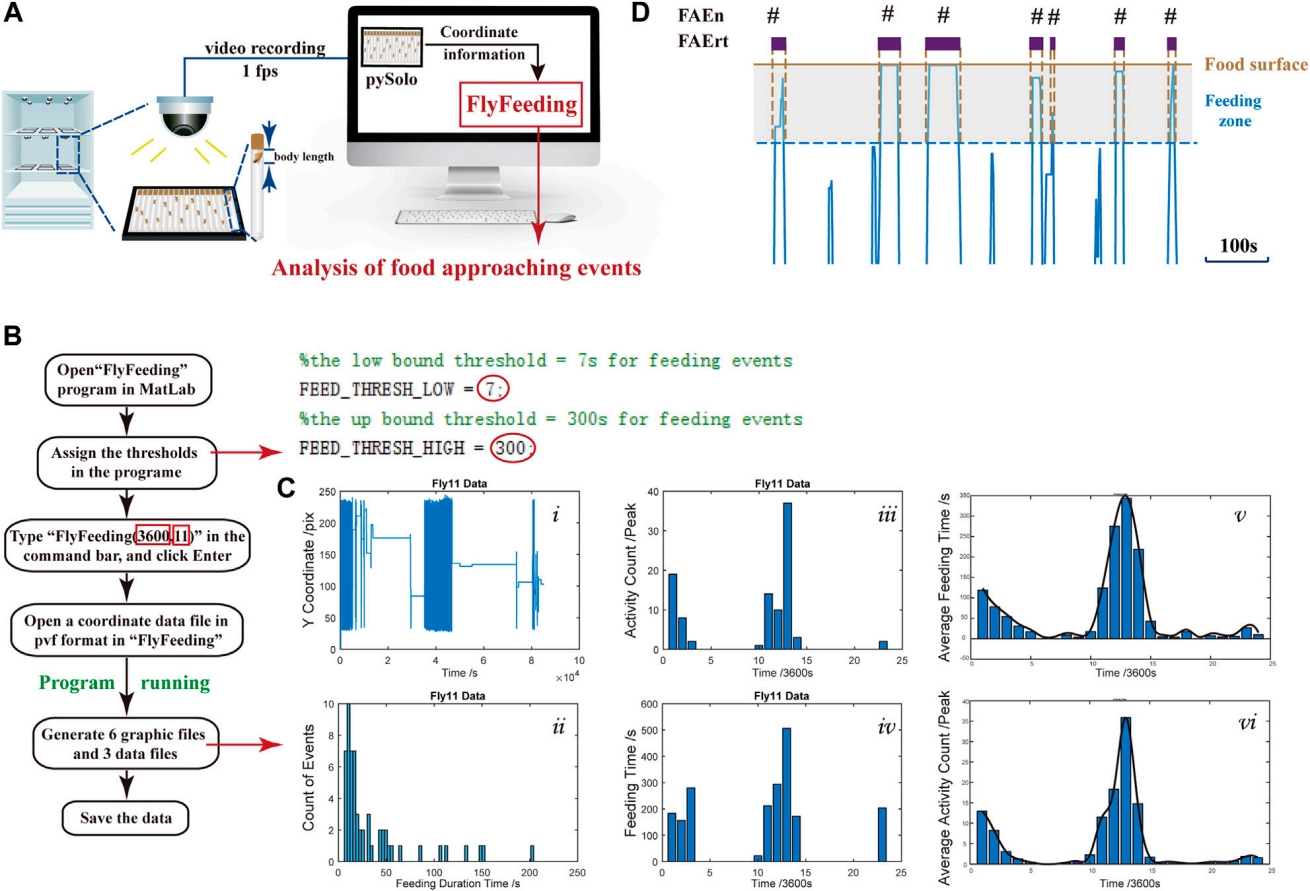
Experimental setup, computer program, and data output **(A)** Experimental setup for dFRAME method. A group of 18 flies were monitored individually and the coordinate data are used for analysis **(B)** Operational flowchart of the “FlyFeeding” program. The right part represents a screenshot of the program start; the cut-off thresholds can be assigned by users (red circles). The two numbers in red rectangles indicate the analyzing period (3,600 s) and fly body length (11 mm) **(C)** “FlyFeeding” simultaneously analyzes a group of 18 flies, generating a set of six graphs and three data flies. Graphs i–iv show the analytical results of a representative fly, including the movement tracks (i), the FAEn distribution according to either the residence time (ii) or in every hour of the day (iii), and the FAErt distribution in every hour of the day (iv). The averaged distribution of FAEn and FAErt of the 18 flies are shown in every hour of the day (v and vi, respectively). In addition, the program generates three data files, “Activity.csv” for FAEn every hour, “Feeding.csv” for total FAErt every hour, and “FeedingPDF.csv” for FAErt of each time. All data are listed for individual flies **(D)** Schematic diagram of food-approaching events (FAE). The brown line indicates the food surface. The gray area represents a fly body length from the food surface, which is determined as feeding zone. A short period of movement track was clipped out, and those close to the food end are shown in blue lines. Once the fly enters the zone, the FAEn counts 1, and the duration that the fly stays in the zone is recorded as the FAErt.

To monitor feeding behavior at a long-term range, we utilized the video recording system, and on each recording plate, 18 flies in 18 glass tubes were recorded. The coordinate data of each fly were obtained using pySolo for further analysis (Figure 1A). To characterize the feeding behavior, we developed a MATLAB program, FlyFeeding (see Figure 1B for the analysis flow chart), allowing synchronous data processing for 18 flies. As shown in Figure 1Ci), the movement track of one representative fly exhibited conspicuous rhythmicity of locomotor activity, shown as intensive track lines around light–dark and dark–light switch periods. Notably, flies did not stay on food for a long period. Instead, flies usually approached the food, ate, left, and returned (Figure 1D), namely, food-approaching behavior.

To quantify the food-approaching behavior, we defined a feeding zone as the distance of one fly body length from the food surface (Figure 1D). In our system, one body length of female flies is approximate 12 pixels and 11 pixels for males (Supplementary Table S1). For each tube, the food surface is automatically detected by the find-peaks module in FlyFeeding, defined as the farthest coordinate position of the fly in every 24-h recording. As shown in Figure 1D, once the fly enters the zone, the FAEn counts 1, and the duration that the fly stays in the zone is recorded as the FAErt.

FlyFeeding provided the distribution of food-approaching events according to their time duration for individual flies, and we found that most events were short in duration (Figure 1Cii). In addition, both numbers and residence time of FAE were high in the morning and the evening periods (Figure 1Ciii-iv). Flies showed high levels of both feeding and locomotor activities in these two periods; thus, we speculated that the FAE were closely related to feeding behavior and also included no-feeding activities.

### Setting Cut-Off Filters to Minimize the Inference of No-Feeding FAE

**FIGURE 2:**
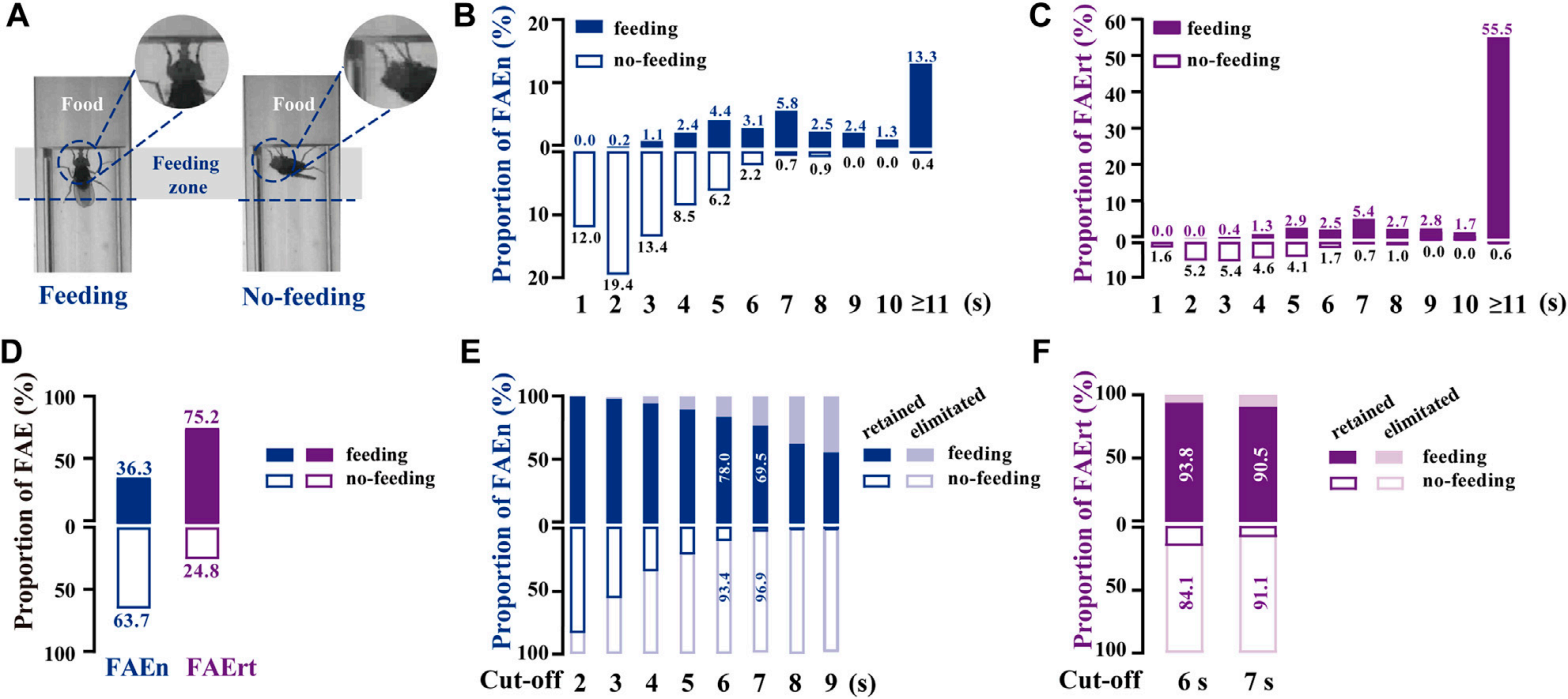
Analyzing food-approaching behavior using high-magnification recording **(A)** Two video snapshot images showing FAE under feeding **(left)** and no-feeding conditions **(right)** **(B)** Proportional distribution of FAEn. The numbers on each column indicate the percentage of the event numbers with or without feeding in the total event number (551) **(C)** Proportional distribution of FAErt. The numbers on each column indicate the percentage of the residence time with or without feeding in the total residence time (4,132 s) **(D)** The proportion of total feeding and no-feeding events (FAEn) and time (FAErt) **(E)** As the cut-off threshold increases from 2 to 9 s, the ratio of retained feeding events to all feeding events (200) gradually decreases, while the ratio of retained no-feeding events to all no-feeding events (351) decreases rapidly **(F)** The proportion of retained FAErt with the cut-off threshold set to either 6 or 7 s.

To distinguish FAE with feeding and with no-feeding, we established a high-magnification video recording system. Similar to our analysis of the regular video recordings, we marked a feeding zone of one body length from the food surface in these high-magnification videos (Figure 2A). Feeding behavior was distinguished manually when flies extended proboscis twice or more times in one FAE. All recorded FAE were classified into two types, namely, food approaching with feeding or with no-feeding (Figure 2A; Supplementary Movie S1, S2).

We found that among 551 FAE collected from 45 male flies, most no-feeding events occurred when the residence time was shorter than 7 s, whereas most feeding events happened when the residence time was longer than 3 s (Figures 2B,C). Notably, food-approaching events lasting longer than 10 s contributed more than half of the residence time, and most of them were accompanied with feeding behavior. Overall, approximately 64% of FAEn and 25% of FAErt were not related to feeding (Figure 2D). We next recorded 634 FAE from 58 female flies. The proportion of feeding and no-feeding FAE exhibited identical profiles to those in males (Supplementary Figure S1A, B). In addition, approximately 65% of events and 30% of residence time were not related to feeding in females (Supplementary Figure S1C).

To minimize the inference of no-feeding FAE, the low cut-off filter was set with the criteria of eliminating the no-feeding events and retaining the feeding events. As shown in Figure 2E, the ratio of retained no-feeding events rapidly declined when the cut-off threshold increased, while the ratio of the retained feeding events decreased at a slower rate. When the low cut-off threshold was set as 7 s, approximately 97% of events and 91% of residence time with no-feeding were not included for calculation, while 70% of events and 91% of residence time with feeding were retained (Figures 2E, F). We next tested the 7-s cut-off in female flies. Approximately 95% of events and 86% of the residence time with no-feeding were rejected, while 77% of the events, together with 93% of residence time with feeding, were retained (Supplementary Figure S1D, E). Therefore, the FAErt with the 7-s cut-off commendably represents feeding status in both female and male flies.

**FIGURE 3:**
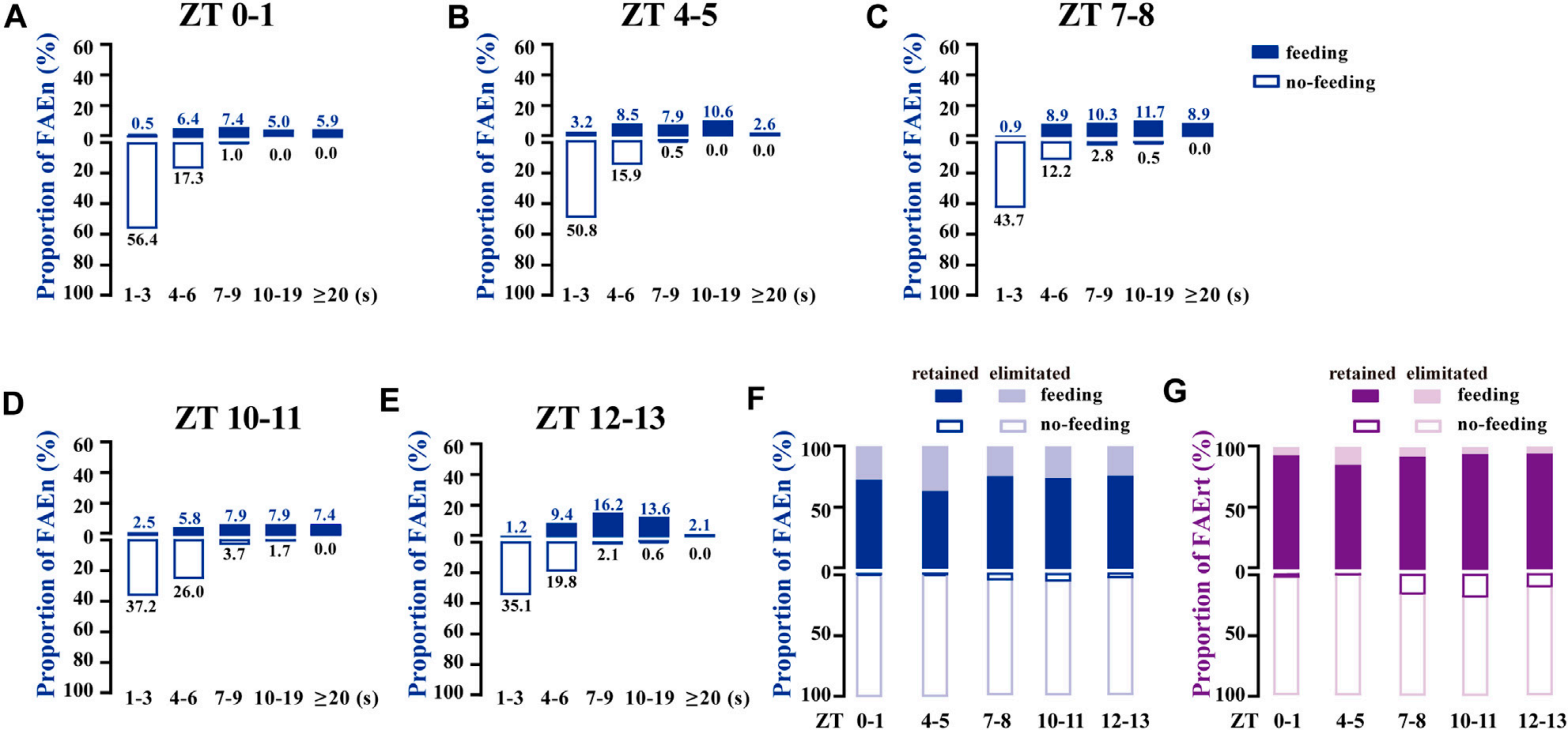
FAE exhibits similar distributions of feeding and no-feeding behaviors at different time-points of the day **(A–E)** The proportion distribution of FAE at ZT0–1 (*n* = 202), ZT4–5 (*n* = 189), ZT7–8 (*n* = 213), ZT10–11 (*n* = 242), and ZT12–13 (*n* = 339) **(F–G)** With the cut-off threshold of 7 s, the proportion of retained and eliminated FAEn **(F)** and FAErt **(G)** at different times of the day. Data are shown for both sexes, with a total of 1,185 events and a total time of 8,603 s used for analysis.

To determine whether the 7-s cut-off is suitable to process the data from different time-points of the day, the proportional distribution of a total of 1,185 FAE was analyzed according to the five recording periods. As shown in Figures 3A–E, most of the no-feeding FAE were shorter (1–3 s) in the morning (ZT 0–1 and 4–5), while the proportion of 4–6 s or longer time increased in late afternoon and evening (ZT 10–11 and 12–13). Nevertheless, these subtle differences failed to affect the choice of the 7-s cut-off. For all five sets of data at different time points, at least 92% of FAEn with no-feeding were eliminated, and 85% of FAErt with feeding were retained with the 7-s cut-off (Figures 3F,G). Together, these results indicated that data collected at different time-points were suitable for processing using the same low cut-off threshold.

When we analyzed the high-magnification video data, we observed six long-lasting FAE events, which were longer than 120 s, with the longest event lasting 250 s. In addition, we identified a small number of FAE with durations longer than 300 s in regular video recording. Because 5 min or more of quiescence is defined as sleep in flies (Shaw et al., 2000; Zimmerman et al., 2008), we set 300 s as the high cut-off threshold. On the basis of these results, we set the band filter of 7–300 s for all further analysis of data obtained from regular video recording for all time-points of the day, for both females and males.

### Validation of the Cut-Off Thresholds in the Regular Video Recording System

**FIGURE 4:**
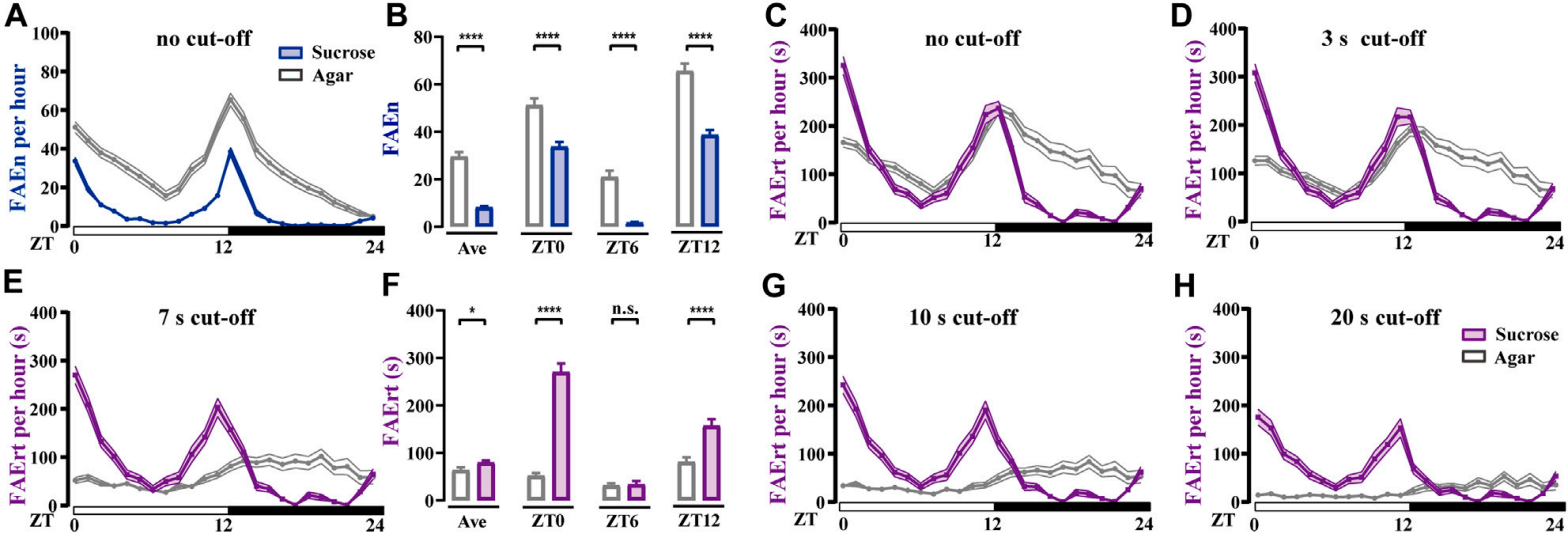
Food-approaching behavior exhibits different profiles in wild-type *w* ^*1118*^ male flies **(A)** Flies in the agar group display higher levels of FAEn compared to FAEn in flies in the sucrose group. The numbers of FAE were merged every hour and are shown in curves **(B)** The average FAEn per hour of the whole day and the FAEn in ZT 0, 6, and 12 **(C–E, G–H)** The curves of FAErt with either no cut-off **(C)** or with cut-off as indicated **(F)** The average FAErt per hour of the whole day and the FAErt in ZT 0, 6, and 12. Data are shown as means ± SEM. *p < 0.05; ****p < 0.0001. n. s. indicates no significant difference. *n* = 78 in the agar group and 86 in the sucrose group.

Fruit flies display significant daily rhythms, showing as a typical double-peak curve with high levels of locomotor activity in the morning and evening (Helfrich-Forster, 2000; Hall, 2003; Schabler et al., 2020; Cascallares et al., 2018). Earlier studies also reported that flies consume food mostly during these time periods (Ro et al., 2014). To test whether our method captures the rhythm of feeding rather than locomotor activity, we video-recorded wild-type *w* ^*1118*^ male flies using two types of foods, either agar alone or agar containing sucrose. As shown in Figure 4A, FAEn exhibited the typical double-peak curve in both groups, indicating that flies approach food more frequently in the mornings and evenings. In addition, we detected significantly more FAE in the agar group compared to events recorded for the sucrose group throughout the day (Figures 4A,B). This result is in agreement with previous findings that hungry flies exhibit a higher locomotor activity (Yang et al., 2015).

We next analyzed the index of FAErt with different cut-off thresholds. We found that the sucrose group exhibited typical double-peak curves regardless of the thresholds (Figures 4C–H). In contrast, the FAErt curves of the agar group turned flatter as the threshold increased, and the peak shape was completely lost when the threshold was 7 s or higher (Figures 4E–H). Moreover, the average FAErt per hour of the agar group was significantly lower than that of the sucrose group, which was more evident in the morning and evening (ZT 0 and ZT 12, Figure 4F). Together with the FAEn results (Figures 4A, B), these findings indicated that when provided with food lacking nutrients, flies display more short-time food-approaching behavior, presumably representing the processes of exploring and foraging. Therefore, the influence of these no-feeding events on the FAErt is effectively eliminated with the 7-s cut-off filter, and the filtered FAErt reliably represents the feeding status of flies.

## Results

### Analysis of Feeding Rhythms in Wild-type Flies

**FIGURE 5:**
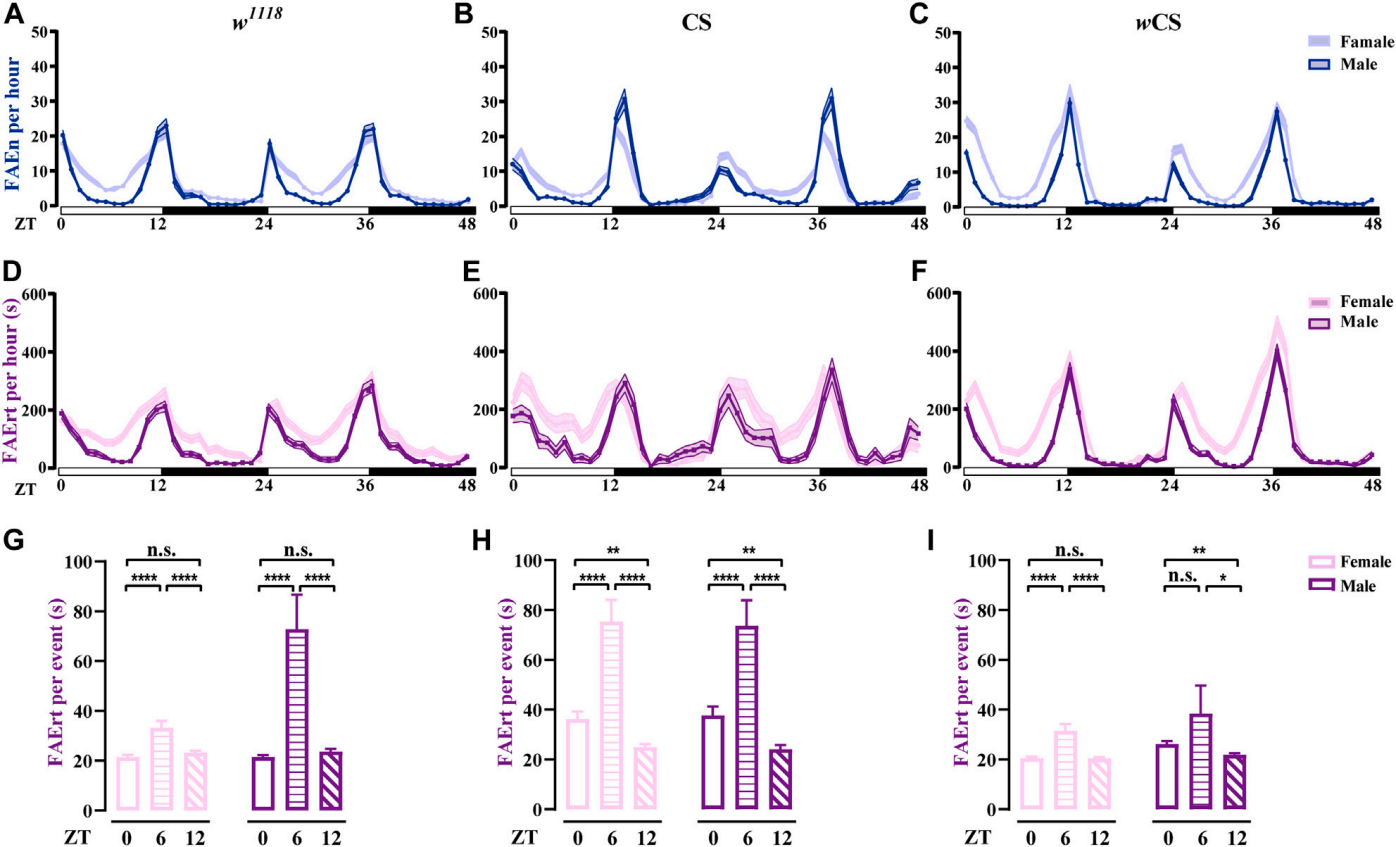
Analysis of feeding rhythms of wild-type flies using dFRAME **(A**–**C)** The FAEn with no cut-off of *w* ^*1118*^ (*n* = 88–89), CS (*n* = 34–36), and *w*CS (*n* = 72–85) flies **(D–F)** The FAErt showing the feeding rhythms of three types of flies. The cut-off threshold is 7–300 s **(G–I)** The FAErt/n showing the average residence time per event of three types of flies. Data are shown as mean ± SEM. *p < 0.05; **p < 0.01; ****p < 0.0001. n. s indicates no significant difference.

Using the dFRAME method with the 7–300-s cut-off, we examined the FAErt of three types of wild-type flies, *w* ^*1118*^, CS, and *w*CS for both female and male flies. As shown in Figures 5A–F, the FAErt of these three strains exhibited the double-peak curve in both sexes, which is in agreement with the results obtained earlier using the FLIC assay (Ro et al., 2014). In addition, our results showed that the FAErt curves of female flies in all three stains were wider in comparison with those in males, indicating longer meal times of female flies (Figures 5D–F). We calculated the FAErt for four time periods, ZT 0–2 (morning peak), 3–11 (day time), 12–14 (evening peak), and 15–23 (night time). Our results supported the observation that compared to male flies, females displayed increased levels of FAErt mostly during day time (Supplementary Figure S2).

Notably, different wild-type fly strains exhibited some unique features. For example, compared to *w* ^*1118*^ and CS, *w*CS displayed sharper peaks in the FAErt curves, especially for males (Figure 5F), indicating that the feeding pattern of *w*CS flies was more concentrated. In addition, CS male flies displayed one-to two-fold more FAEn in the evening compared with that in the morning (Figure 5B); however, the levels of FAErt were close between these two peaks (Figure 5E), indicating that CS flies displayed more short-time no-feeding visiting in the evening. Moreover, the FAErt curves showed a small noon peak in CS male flies, while it did not exist in the FAEn curves.

To better characterize the food-approaching behavior, we analyzed a new parameter, residence time per visit, namely, FAErt/n. Interestingly, although the FAErt levels were lowest in the noon time, the FAErt/n levels were significantly higher than the other two time points in all three fly strains, which was the most evident in CS flies (Figures 5G–I), suggesting that flies take food in a more relaxed manner during lunch time. Notably, the average FAErt/n of female flies were comparable or lower than males for all three time-points, dispelling the concern that the FAErt levels of females were inflated by the egg-laying behavior. Taken together, our dFRAME analytical method characterizes the feeding rhythm of wild-type flies and reveals detailed information about their feeding patterns, thereby allowing cross-comparison among different fly strains.

### Analysis of Feeding Rhythm Under Constant Darkness Condition

**FIGURE 6:**
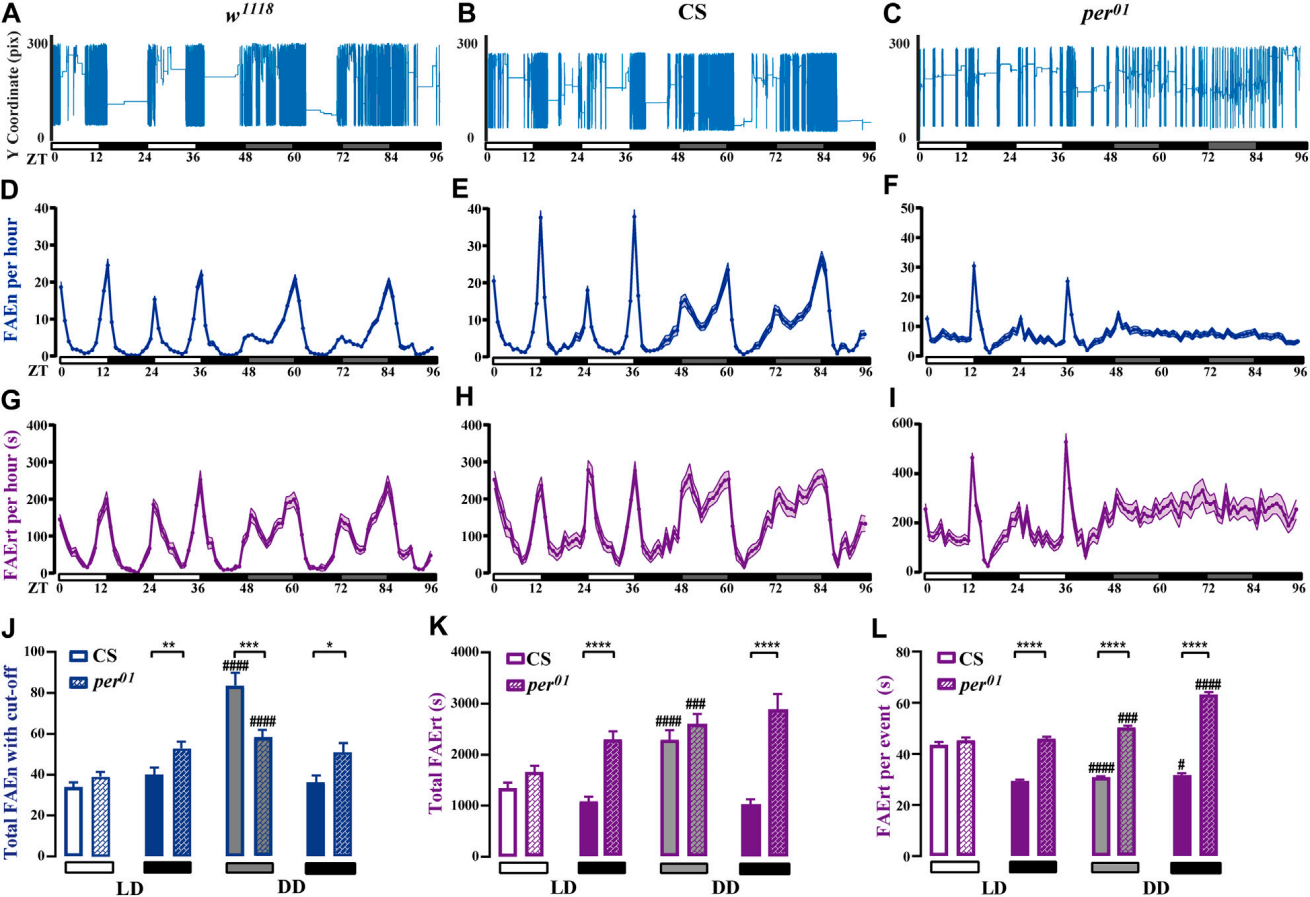
Feeding rhythm analysis under LD and DD conditions of wild-type and *per* ^*01*^ mutant flies **(A–C)** The movement track of a representative male fly of wild-type *w* ^*1118*^ and CS, as well as *per* ^*01*^ mutant **(D–I)** dFRAME analysis of FAEn (**D–F**, with no cut-off) and FAErt **(G–I)** for three types of flies under continuous LD and DD cycles for 4 days **(J–L)** The day time and night time of total FAEn (**J**, with 7-s cut-off), total FAErt **(K)**, and FAErt/n **(L)** under LD and DD conditions. Symbols: #, ##, ###, and #### indicate significance when comparing DD conditions to LD conditions of corresponding genotype and corresponding day or night time. *w* ^*1118*^, *n* = 88; CS, *n* = 66; and *per* ^*01*^, *n* = 72. Data are shown as mean ± SEM. #*p* < 0.05; ** or ##*p* < 0.01; ###*p* < 0.001; **** or ####*p* < 0.0001. No label means no significant difference.

Light is a strong zeitgeber for the circadian rhythm. To study the effects of light on feeding rhythm, we examined *w* ^*1118*^ and CS male flies under LD and DD cycles using our dFRAME method. Compared to LD condition, both wild-type flies displayed higher locomotor activities in the subjective day time under DD condition (Figures 6A, B). Consistently, the FAEn and FAErt also significantly increased in day time, however, not in night time (Supplementary Figure S3A, B, D, E). As shown in Figures 6D, E, G, H, the FAEn and FAErt curves remained in double-peak shape under DD condition, while some changes were evident. For example, the morning peak of the FAEn curve in *w* ^*1118*^ and the evening peak in CS decreased, whereas the FAErt peak values remained stable; moreover, the peak width in both FAEn and FAErt curves increased under DD condition. These findings indicated that in the absence of light, wild-type flies are able to maintain the 24-h cycle of feeding rhythm, as well as the levels of feeding behavior at morning and evening meal times; however, they elicit more food-approaching behavior in non-meal day time, so that the concentrated feeding pattern that is observed under LD condition is weakened.

It has been reported that *per* ^*01*^ mutant flies display rhythmic feeding patterns in LD cycles; however, this pattern is lost under a DD condition (Ro et al., 2014; Schabler et al., 2020). In agreement with these observations, we found that *per* ^*01*^ flies displayed daily feeding rhythms under LD condition. However, our dFRAME method showed that both the FAEn and the FAErt curves of *per* ^*01*^ flies exhibited only one peak in the evening, with the morning peak inapparent, which was different from the typical double-peak curves observed in wild-type flies (Figures 6D–I). In addition, wild-type flies started to approach food more frequently before light was off, whereas the food-approaching behavior of *per* ^*01*^ flies did not increase until the light was off, suggesting that loss-of-function of *period* impairs the organization of morning feeding and the prediction of light off.

When the light condition was switched to DD, *per* ^*01*^ flies completely lost their feeding rhythm. As shown in Figures 6F, I, both the FAEn and FAErt curves remained flat throughout the day. Similar to the genetic control CS flies, the FAEn in the subjective day time also increased in *per* ^*01*^ flies (Supplementary Figure S3B, C); however, the increase occurred at a significantly lower level compared with CS flies (Figure 6J). In contrast, we found that the FAErt levels in subjective day time of *per* ^*01*^ flies increased to a similar level of those in CS flies (Figure 6K). Two-way ANOVA showed that the DD condition affected the day time FAEn differently between these two fly strains (*p* < 0.001) but at similar levels on FAErt (*p* > 0.05). We thus calculated the average food residence time per event (FAErt/n). Intriguingly, when switched to the DD condition, the day time FAErt/n of CS flies significantly decreased, whereas it increased in *per* ^*01*^ flies (Figure 6L), with a highly significant interaction between genotypes and light conditions (*p* < 0.0001, two-way ANOVA).

For the night time, the FAEn levels of *per* ^*01*^ flies were comparable between the LD and DD conditions, and they were modestly higher than those of CS flies (Figure 6J). Two-way ANOVA indicated that the *p* value was greater than 0.05. However, *per* ^*01*^ flies displayed significantly higher FAErt levels than CS flies in night time of LD cycles (Figure 6K), and the difference was enlarged under the DD condition (*p* < 0.05, two-way ANOVA). Consequently, the average FAErt/n during night time were significantly longer in *per* ^*01*^ flies than those in CS flies under both LD and DD conditions (Figure 6L), and the difference was more evident under the DD condition (*p* < 0.0001, two-way ANOVA).

Taken together, our findings indicate that under the LD condition, *per* ^*01*^ mutant flies respond to the lights off to maintain daily feeding rhythms. They visit food sites (FAEn) at comparable levels to CS control flies, however, stay longer (FAErt/n and FAErt) in the night than CS flies. Under the DD condition, both wild-type and mutant flies perform more food-approaching behavior during the subjective day time but with different feeding patterns. CS flies visit food more often with shorter residence time per stay, whereas mutant flies prolong their residence time every visit, similar to that in night time under the LD condition.

## Discussion

Here, we report a video recording-based analytic method called dFRAME for studying the feeding rhythm in *Drosophila*. Utilizing the high-magnification video recording system, we determined a cut-off range of 7–300 s and three indexes, the numbers of FAE (FAEn), the residence time (FAErt), and the average residence time per event (FAErt/n), to analyze feeding patterns. Our method recaptured the feeding rhythms earlier found in these flies and, moreover, revealed new profiles of feeding behavior.

*Drosophila* is a powerful genetic animal model to investigate the molecular and neural mechanisms underlying behaviors. Several assays have been developed to analyze feeding behavior at different time ranges, including PER for testing immediate response (within 10 min), tracer-based methods for short-term examination (0.5–1 h), electric signal-based methods (Fly Pad and FLIC) for medium-term recording (1–3 days), and the video-based method (ARC) for long-term monitoring (up to 7 days). Our dFRAME method is also a video-based method, and the recording is even longer (up to 1–2 weeks) and more stable than ARC, because no liquid food is used in our system. The advantage of ARC is that it reports the precise levels of food intake; however, the temporal resolution of 1 min makes it difficult to capture the detailed information of feeding behaviors. In contrast, the data obtained by dFRAME are less correlated to food consumed; instead, they characterize feeding-related behavioral features in a highly reproducible manner. For studying feeding rhythms in a long term, dFRAME is ideal to perform high-throughput screen and explore new behavioral patterns, and ARC can be used to determine the amount of food intake.

dFRAME is built on the basis of video-recording system that has been used for analyzing sleep. Thus, we are able to analyze locomotor activity, foraging, feeding, and sleep from the same set of video data. In addition, since the infrared light is used for video recording, the light conditions (e.g., LD, DD, and LL) can be designed and conducted without interfering the recording. Similarly, genetical methods for manipulating neural activity, including heat and optogenetics controls of target neurons, can also be incorporated into the system. Moreover, the fly chamber can be modified according to experimental requirements. In this study, we use standard straight glass tubes with food in one end. It is also applicable to two-end or multi-end chambers by adding modules in the FlyFeeding program. Electrically operated gates can also be integrated. Therefore, taking the advantage of powerful genetic manipulation and rich behavior of *Drosophila*, dFRAME enables a comprehensive investigation of feeding rhythms.

In this study, we tested *per* ^*01*^ mutant flies, which has been reported to exhibit feeding rhythms under the LD condition (Ro et al., 2014; Schabler et al., 2020). Consistently, we found that these flies display 24-h rhythms; however, our results indicated that they fail to predict the timing for the evening meal and almost completely lose the breakfast even under LD cycles. Intriguingly, during the subjective day time under the DD condition, both *per* ^*01*^ flies and the genetic control CS flies increase their total food residence time to a similar level; however, they achieve this using two different approaches. Specifically, the *per* ^*01*^ mutant flies considerably prolong the residence time per stay (FAErt/n) to the similar levels observed during the night. In comparison, CS flies dramatically decrease the residence time per stay and instead revisit the food more frequently. These findings suggest that CS flies are aware of the day-time period in DD cycles and the cognitive dissonance results in anxiety-like behavior. This is in agreement with a previous finding that CS flies display decreased day-time sleep under DD conditions (Parisky et al., 2016). In contrast, lacking of the internal clock, *per* ^*01*^ mutant flies exhibit same feeding patterns in the subjective day time to that observed during the night. These interesting profiles identified through our dFRAME analysis await further mechanistic investigation in future.

## Summary Statement

We developed a new analytical method, dFRAME, which is simple, stable, robust, and reliable for investigating the feeding rhythms of fruit flies.

## Data Availability

The raw data supporting the conclusions of this article will be made available by the authors, without undue reservation.
