# Supplementary material for: dFRAME: A Video Recording-Based Analytical Method for Studying Feeding Rhythm in Drosophila
Source: Front Genet. 2021 Oct 15;12:763200. doi: 10.3389/fgene.2021.763200 (PMC8554052; doi:10.3389/fgene.2021.763200)
Supplement: Supplementary file 3 [file DataSheet1.docx]

**dFRAME: A video recording-based** **analytical method for studying feeding rhythm in *Drosophila***

**Supplementary Figures**


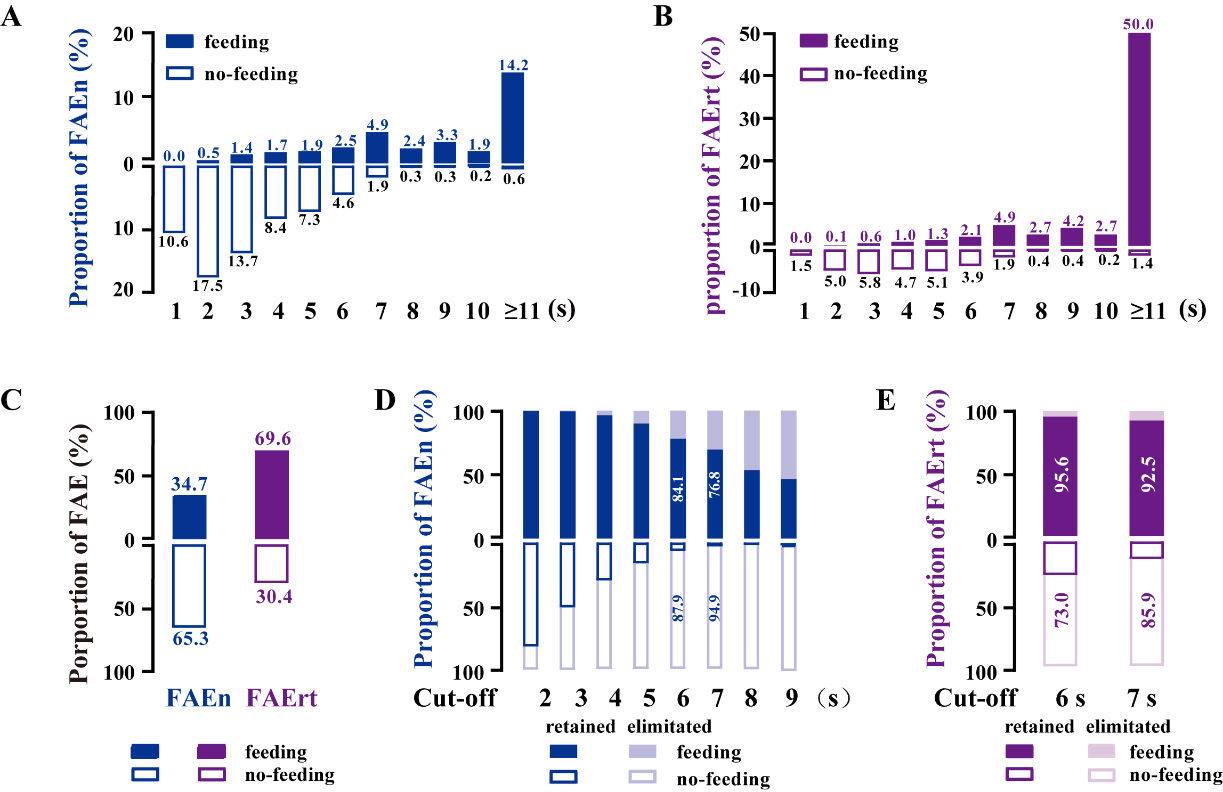


**Supplementary Figure 1. Profile of FAEn and FAErt in *w^1118^* female flies.**

1. Proportional distribution of FAEn. The numbers on each column indicate the percentage of the event numbers with or without feeding in the total event number (634).
2. Proportional distribution of FAErt. The numbers on each column indicate the percentage of the residence time with or without feeding in the total residence time (4471 s).
3. The proportion of total feeding and no-feeding FAEn and FAErt.
4. As the cut-off threshold increases from 2 to 9 s, the ratio of retained feeding events to all feeding events (220) gradually decreases, while the ratio of retained no-feeding events to all no-feeding events (414) decreases rapidly.
5. The proportion of retained FAErt with the cut-off threshold set to either 6 or 7 s.


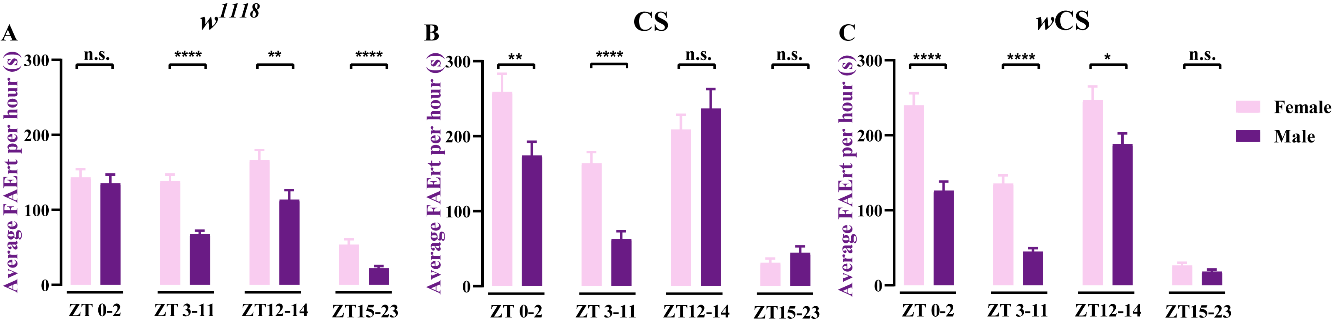


**Supplementary Figure 2. Average FAErt during different time periods of the day for three wild-type fly strains.**

Data are shown as mean± SEM. *, *p*＜0.05; **, *p*< 0.01; ****, *p*< 0.0001. n.s. indicates no significant difference. *w^1118^*, n = 88-89.CS, n = 34-36. *w*CS, n = 72-85.


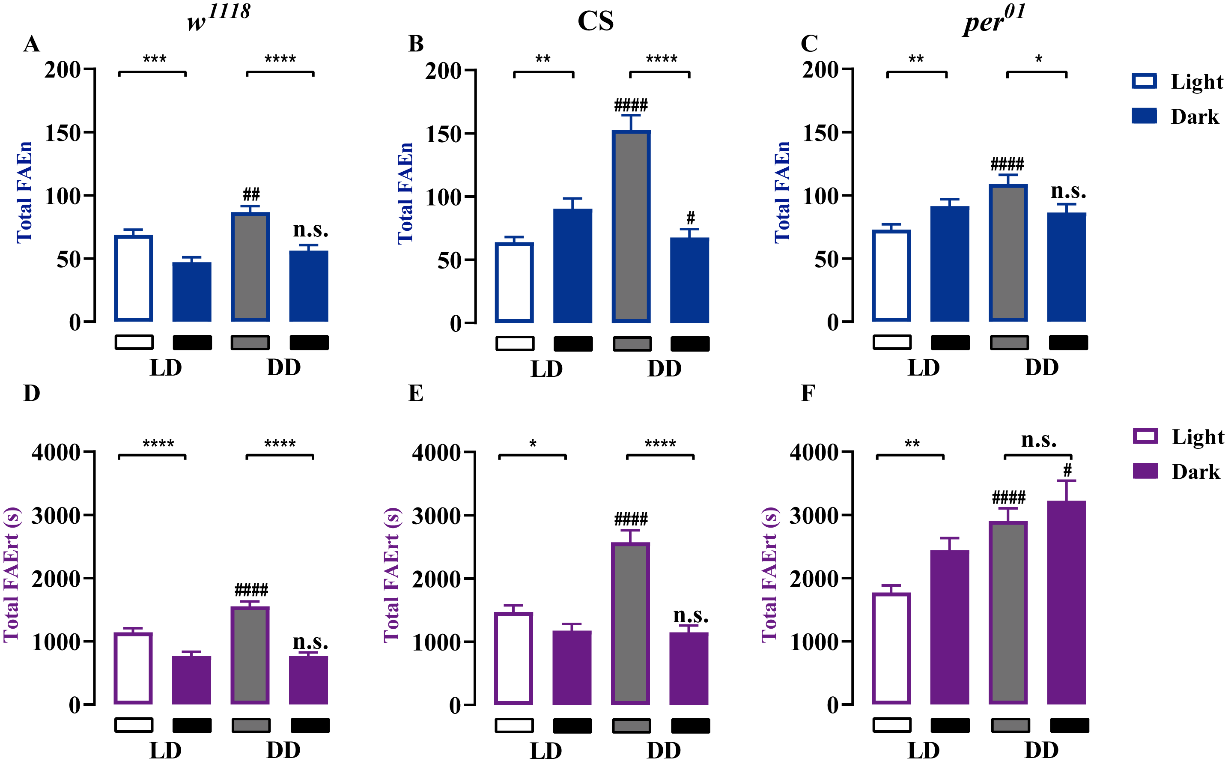


**Supplementary Figure 3. Day-time and night-time total FAEn (with no cut-off) and total FAErt under LD and DD conditions.**

*w^1118^*, n = 88; CS, n = 66; and *per^01^*, n = 72. Data are shown as mean± SEM. * or #, *p*＜0.05; **or ##, *p*< 0.01; ***, *p*< 0.001; ****or ####, *p*< 0.0001. ns, no significant difference. Symbol #, ##, and #### indicate the significance when comparing DD condition to LD condition of corresponding genotype and corresponding day-time or night-time.

**Supplementary Table 1. Body length of different fly stains.**

| Genotype | Female | | Male | |
| --- | --- | --- | --- | --- |
|  | mm | pixel | mm | pixel |
| *w^1118^* | 2.67±0.05 | 12.3±0.2 | 2.34±0.02 | 10.8±0.1 |
| CS | 2.77±0.01 | 12.7±0.1 | 2.41±0.01 | 11.1±0.1 |
| *w*CS | 2.78±0.01 | 12.8±0.1 | 2.44±0.02 | 11.2±0.1 |
| *per^01^* | 2.70±0.02 | 12.4±0.1 | 2.42±0.01 | 11.1±0.1 |

n = 24 for each group.

**Legends for Supplementary Movies**

Supplementary Movie 1. A representative food approaching event with feeding observed from a *w^1118^* female fly.

Supplementary Movie 2. A representative food approaching event with no feeding observed from a *w^1118^* female fly.
